# Genomic Reclassification and Phenotypic Characterization of *Pseudomonas putida* Strains Deposited in Japanese Culture Collections

**DOI:** 10.1264/jsme2.ME23019

**Published:** 2023-06-06

**Authors:** Tomohiro Morohoshi, Naoya Yaguchi, Nobutaka Someya

**Affiliations:** 1 Graduate School of Regional Development and Creativity, Utsunomiya University, 7–1–2 Yoto, Utsunomiya, Tochigi 321–8585, Japan; 2 Institute for Plant Protection, National Agriculture and Food Research Organization (NARO), 2–1–18 Kannondai, Tsukuba, Ibaraki 305–8666, Japan

**Keywords:** *Pseudomonas putida*, comparative genome, orthologue, quorum sensing, reclassification

## Abstract

*Pseudomonas putida* is a major species belonging to the genus *Pseudomonas*. Although several hundred strains of *P. putida* have been deposited in culture collections, they potentially differ from the genetically defined “true *Pseudomonas putida*” because many were classified as *P. putida* based on their phenotypic and metabolic characteristics. A phylogenetic ana­lysis based on the concatenated sequences of the 16S rRNA and *rpoD* genes revealed that 46 strains of *P. putida* deposited in Japanese culture collections were classified into nine operational taxonomic units (OTUs) and eleven singletons. The OTU7 strain produces *N*-acylhomoserine lactone as a quorum-sensing signal. One of the OTU7 strains, JCM 20066, exhibited *a ppuI-rsaL-ppuR* quorum-sensing system that controls biofilm formation and motility. The *P. putida* type strain JCM 13063^T^ and six other strains were classified as OTU4. Classification based on the calculation of whole-genome similarity revealed that three OTU4 strains, JCM 20005, 21368, and 13061, were regarded as the same species as JCM 13063^T^ and defined as true *P. putida*. When orthologous genes in the whole-genome sequences of true *P. putida* strains were screened, PP4_28660 from *P. putida* NBRC 14164^T^ (=JCM 13063^T^) was present in all true *P. putida* genome sequences. The internal region of PP4_28660 was successfully amplified from all true *P. putida* strains using the specific primers designed in this study.

*Pseudomonas* is a heterotrophic, motile, Gram-negative, rod-shaped bacterium found in natural environments, such as plants, soil, animals, and water (Palleroni, 1993; Diggle and Whiteley, 2020). The genus *Pseudomonas* was initially described by Migula according to the morphological characteristics of its members (Migula, 1894). The genus *Pseudomonas* was subsequently subdivided into five groups based on the findings of DNA-DNA and rRNA-DNA hybridization (Palleroni *et al.*, 2005). Among these groups, group I is referred to as the genus *Pseudomonas*, which is called “true *Pseudomonas*” or “*Pseudomonas* sensu stricto”, whereas different designations have been proposed for the members of groups II-V (Scarpellini *et al.*, 2004). *Pseudomonas* group I includes many fluorescent and non-fluorescent strains. The most common fluorescent species in *Pseudomonas* group I are *Pseudomonas fluorescens* and *Pseudomonas putida*, which are known as fluorescent *Pseudomonas* (Scarpellini *et al.*, 2004).

*P. putida* is one of the major species of *Pseudomonas* group I, which performs many functions, such as plant growth promotion and bioremediation. For example, *P. putida* F1 is a soil isolate that is capable of chemotaxis for the degradation of numerous aromatic compounds (Hughes *et al.*, 2017). *P. putida* KT2440 induces systemic resistance in plants in response to certain pathogens (Matilla *et al.*, 2010). In addition, strain KT2440 metabolizes a wide range of aromatic compounds and has potential for the environmental bioremediation of industrial waste (Gong *et al.*, 2017). In contrast, some strains of *P. putida* are opportunistic human pathogens responsible for bacteremia, sepsis in neonates, and neutropenia (Horii *et al.*, 2005). Several hundred strains of *P. putida* have been deposited in culture collections worldwide. Many of the *P. putida* strains deposited in these culture collections have been classified as *P. putida* based on their phenotypic and metabolic characteristics, such as morphology, cultural characteristics, and biochemical testing, and not on genetic techniques. Therefore, the classification of these strains potentially differs from *P. putida*. In the present study, we defined the *P. putida* type strain and its genetically related strains as “true *P. putida*”. We reclassified *P. putida* strains deposited in Japanese culture collections based on genomic and phenotypic diversities and developed specific PCR primers to identify true *P. putida* among these bacterial strains.

## Materials and Methods

### Bacterial strains and growth conditions

The *P. putida* strains used in the present study are listed in Table 1. Forty-six strains of *P. putida* were obtained from the Japan Collection of Microorganisms (JCM) or NITE Biological Resource Center (NBRC). *Escherichia coli* was grown in Luria-Bertani (LB) medium at 37°C. *P. putida* strains were grown in trypticase soy broth (TSB; Becton, Dickinson and Co.) or King B medium (KB; Eiken) at 30°C. *Chromobacterium violaceum* CV026 (McClean *et al.*, 1997) and VIR07 (Morohoshi *et al.*, 2008) were grown in LB medium at 30°C. A solid bacterial medium was prepared by adding agar to obtain a final concentration of 1.5%. Antibiotics were added at final concentrations of 100‍ ‍μg mL^–1^ (ampicillin and carbenicillin) and 50‍ ‍μg mL^–1^ (kanamycin), as required. *N*-(3-oxodecanoyl)-l-homoserine lactone (3-oxo-C10-HSL) was synthesized, as previously described (Chhabra *et al.*, 2003). 3-oxo-C10-HSL was dissolved in dimethyl sulfoxide (DMSO) to prepare a 10‍ ‍mM stock solution and added to the medium at a final concentration of 1‍ ‍μM for the biofilm and motility test described below.

### Clustering of concatenated sequences of 16S rRNA and *rpoD* genes

The 16S rRNA and *rpoD* genes were amplified by PCR using Blend Taq Plus DNA polymerase (Toyobo) and the specific primer sets 16SF3/16SR3 (Sawada *et al.*, 2011) and PsEG30F/PsEG790R (Mulet *et al.*, 2010), respectively (Table S1). PCR for the 16S rRNA gene was performed using the following cycling para­meters:‍ ‍94°C for 30‍ ‍s, 50°C for 30‍ ‍s, and 72°C for 1.5‍ ‍min for 30 cycles. PCR for the *rpoD* gene was conducted using the following cycling parameters: 98°C for 10‍ ‍s, 55°C for 30‍ ‍s, and 72°C for 1‍ ‍min for 30 cycles. Sequencing was performed using the BigDye Terminator ver. 3.1 and an Applied Biosystems 3500 Series Genetic Analyzer (Applied Biosystems). A phylogenetic tree based on the‍ ‍concatenated sequences of the 16S rRNA and *rpoD* genes was‍ ‍constructed using the neighbor-joining method with the ClustalW program in MEGA 7 (Kumar *et al.*, 2016). Clustering of the concatenated sequences of the 16S rRNA and *rpoD* genes for operational taxonomic unit (OTU) predictions was performed according to a previously described method (Morohoshi *et al.*, 2018). OTUs were defined with ≥98% identity for clustering ana­lyses.

### Assay for fluorescence production, motility, and antifungal activity

Colonies of *P. putida* strains were formed on TSB agar plates. To assess fluorescence production, colonies of *P. putida* strains were inoculated onto the KB agar plate and incubated at 30°C for two days. Fluorescence was detected after irradiation with ultraviolet (UV) light (λ=365‍ ‍nm). To evaluate motility, colonies of *P. putida* strains were inoculated onto KB soft agar (0.5 wt%) medium. After an incubation at 25°C for 24 h, motility was observed by measuring the spread of colonies on assay plates. To detect antifungal activity, a mycelial disc of *Rhizoctonia solani* MAFF 726551 was placed on top of a dNBYG agar plate (Takeuchi *et al.*, 2014). *P. putida* strains were inoculated at the bottom of dNBYG agar plates. After an incubation at 25°C for three days, antifungal activity was estimated by inhibiting the development of the mycelia of *R. solani*.

### Identification of *N*-acylhomoserine lactone (AHL) molecules

AHL production was tested by cross-streaking two AHL reporter strains, *C. violaceum* CV026 and VIR07 (Morohoshi *et al.*, 2021). CV026 and VIR07 were streaked horizontally on LB agar plates, while *P. putida* strains were streaked vertically next to them. After an overnight incubation at 30°C, AHL produced by the strains under study diffused and induced the production of the purple pigment violacein by AHL reporter strains. AHL-producing activity was evaluated on three levels (strong, weak, and none) based on the intensity of the purple color. AHL molecules produced by *P. putida* were extracted from the culture supernatant using a previously described method (Morohoshi *et al.*, 2019). AHL molecules were identified using Liquid Chromatography-Tandem Mass Spectrometry (LC-MS/MS), as previously described (Morohoshi *et al.*, 2019).

### Whole-genome shotgun sequencing

The genomic DNA of *P. putida* strains was extracted using a‍ ‍standard protocol with sodium dodecyl sulfate (SDS) and proteinase K. Library construction and sequencing using the Illumina NovaSeq 6000 and HiSeq X Ten platforms were performed using the commercial services of Eurofins Genomics. Adapter-trimmed raw reads were assembled using SPAdes v3.13.0 (Bankevich *et al.*, 2012). Assemblies were annotated using the DDBJ Fast Annotation and Submission Tool (DFAST) version 1.2.18, which is a bacterial genome annotation pipeline (Tanizawa *et al.*, 2018). Briefly, coding sequences (CDSs) were predicted using MetaGeneAnnotator (Noguchi *et al.*, 2008). Genes coding for tRNA and rRNA were identified using Aragorn 1.2.38 (Laslett and Canback, 2004) and Barrnap 0.8 (https://github.com/tseemann/barrnap), respectively.

### Comparative genome ana­lysis

The whole genome sequences of *P. putida* were retrieved from sequences deposited in the NCBI genome website (https://www.ncbi.nlm.nih.gov/genome) on Jan 20, 2023. Whole-genome similarity was analyzed by calculating average nucleotide identity (ANI) and digital DNA-DNA hybridization (dDDH) values. ANI values were calculated by the pairwise calculation method using the OrthoANIu online tool (https://www.ezbiocloud.net/tools/ani) (Yoon *et al.*, 2017). dDDH values were calculated using formula two of the Genome-to-Genome Distance Calculator 3.0 (https://ggdc.dsmz.de/ggdc.php) (Meier-Kolthoff *et al.*, 2022). Orthologous genes among *P. putida* strains were inferred using OrthoFinder 2.5.4 (Emms and Kelly, 2015). To detect the presence of one orthologous gene, PP4_28660, the internal region of PP4_28660 was amplified by direct colony PCR using the KOD One PCR Master Mix (Toyobo) and the specific primer sets, Ppu28660-F/Ppu28660-R (Table S1). PCR was performed using the following cycling parameters: at 98°C for 10‍ ‍s, 60°C for 5‍ ‍s, and 68°C for 30‍ ‍s for 30 cycles.

### Cloning and disruption of quorum-sensing genes

The *ppuI* and *ppuR* coding regions in the JCM 20066 genome were amplified using Blend Taq Plus DNA polymerase and specific primers (Table S1). PCR products were cloned into the pGEM-T Easy Cloning Vector (Promega). To remove the internal sequences of the target genes, sequences upstream and downstream of the target gene were amplified using pGEM-T-containing plasmids as the template and specific primers for gene deletion (Table S1). Amplified PCR fragments were excised via *Bam*HI digestion and self-ligated. The gene-coding region with a deletion in the internal sequence was excised using *Eco*RI and inserted into the *Eco*RI site of the suicide vector pK18mobsacB (Schafer *et al.*, 1994). Deletion mutants of quorum-sensing genes were generated by homologous recombination using a previously described method (Morohoshi *et al.*, 2022).

### Biofilm formation assay

Overnight cultures of JCM 20066 and its mutants were diluted to an OD_600_ of 0.01 in TSB medium. One hundred microliters of this dilution was transferred to a 96-well polystyrene plate (AsOne). After an incubation at 30°C for 18 h, 25‍ ‍μL of 1% crystal violet solution was added to each well. The plates were then incubated at room temperature for 15‍ ‍min and rinsed twice with distilled water. Crystal violet was dissolved in 100‍ ‍μL of 99.5% ethanol. Biofilm formation was quantified by measuring absorbance at 570‍ ‍nm (A_570_) using an Infinite M200 microplate reader (Tecan Japan). The A_570_ values of the wells were averaged, and standard deviations were calculated. Means were separated using Tukey’s honest significant difference (HSD) test (*P*<0.01).

### Nucleotide sequence accession number

The whole genome shotgun sequences of *P. putida* were deposited in the DDBJ/ENA/GenBank databases under the accession numbers listed in Table 2. The raw sequencing reads used in this study have been deposited in the DDBJ Sequence Read Archive under the accession number DRA015616. The nucleotide sequences of 16S rRNA and *rpoD* reported in this study were deposited in the DDBJ/ENA/GenBank databases under the accession numbers listed in Table S2.

## Results and Discussion

### Phylogenetic ana­lysis of *P. putida* strains

**Fig. 1.**
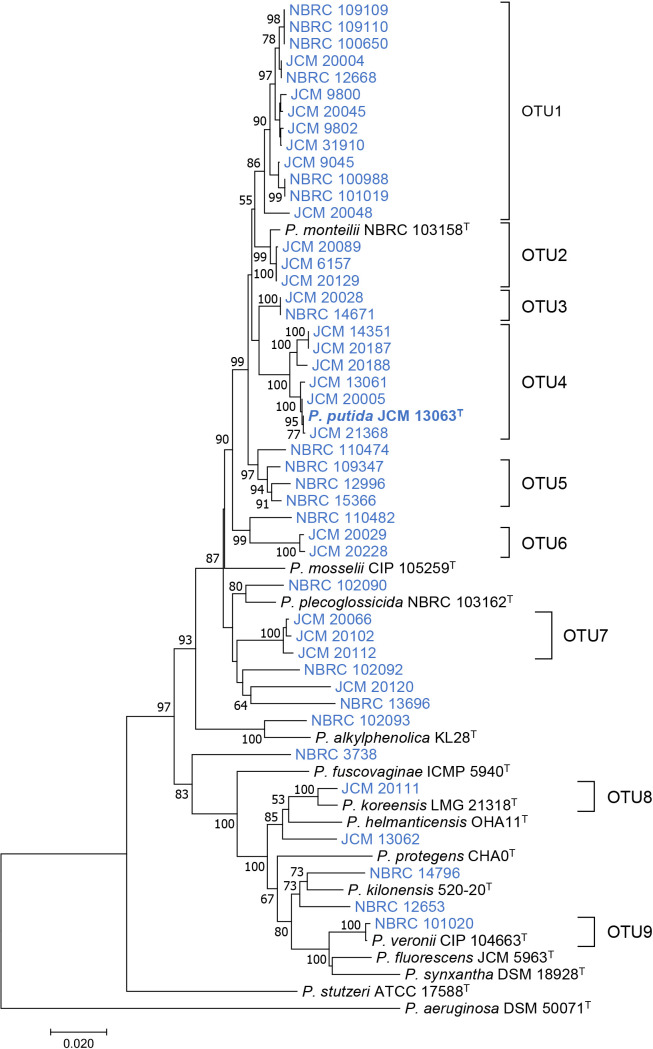
Phylogenetic tree based on concatenated sequences of 16S rRNA and *rpoD* genes from *Pseudomonas putida* strains (blue) and type strains of other *Pseudomonas* species (black). The *P. putida* type strain JCM 13063^T^ is shown in bold. The phylogenetic tree was constructed by the neighbor-joining method using the ClustalW program of the MEGA 7 program. The percentage of replicate trees in which the associated taxa clustered together in the bootstrap test (500 replicates) are shown next to the branches. The scale bar represents 0.02 substitutions per nucleotide position.

The 16S rRNA genes of 46 strains of *P. putida* and 14 type strains of other species of *Pseudomonas* were sequenced and used for an OTU clustering ana­lysis. When the OTUs were strictly defined based on ≥99% identity, 39 strains of *P. putida* and type strains of *P. mosselii*, *P. monteilii*, and *P. plecoglossicida* were classified into the same OTU (Fig. S1). These results indicate that 16S rRNA sequences were not suitable for the classification of *P. putida* and other *Pseudomonas* species. Therefore, another housekeeping gene, *rpoD*, was sequenced and used for OTU clustering. The phylogenetic tree based on *rpoD* sequences showed more diverse branching than that based on 16S rRNA sequences (Fig. S2). Based on these results, concatenated sequences of the 16S rRNA and *rpoD* genes were used for OTU clustering for a more accurate classification of *P. putida* strains. A phylogenetic tree based on the concatenated sequences of the 16S rRNA and *rpoD* genes is shown in Fig. 1. Clustering of the concatenated sequences of the 16S rRNA and *rpoD* genes at 98% identity revealed that 46 strains of *P. putida* were classified into nine OTUs and eleven singletons. Strains classified as OTU2, OTU4, OTU8, and OTU9 showed high identity with type strains *P. monteilii*, *P. putida*, *P. korrensis*, and *P. veronii*, respectively. Since the other strains did not show a certain degree of identity with the type strains of *Pseudomonas* species, these strains may be classified as novel *Pseudomonas* species or subspecies. The* P. putida* type strain JCM 13063^T^ was classified as OTU4, which includes only six strains: JCM 13061, JCM 14351, JCM 20005, JCM 20187, JCM 20188, and JCM 21368. Therefore, only these seven strains were classified as true *P. putida*.

### Phenotypic characterization of *P. putida* strains

Regarding classification based on physiological differences, various typical phenotypes were compared among *P. putida* strains. *P. putida* belongs to a wide group of fluorescent *Pseudomonas* species (Scarpellini *et al.*, 2004). The fluorescence of *P. putida* strains was assayed using KB agar medium. Most strains produced fluorescence; however, a few non-fluorescent strains were scattered despite their OTU classification (Table 1). Motility was assayed using a KB plate containing 0.5 wt% agar. Although 26 strains of *P. putida* showed motility, the presence or absence of motility was not related to the OTU classification (Table 1). Some *P. putida* strains have been shown to inhibit the growth of plant pathogenic fungi (Bagnasco *et al.*, 1998). Therefore, the antifungal activities of the 46 *P. putida* strains were assayed by cross-streaking them against the plant pathogen *R. solani*. However, only two strains, NBRC 109347 and NBRC 14796, exhibited antifungal activity (Table 1). One antifungal strain, NBRC 14796, was phylogenetically different from the other *P. putida* strains and was related to *P. protegens*, which is a well-known biocontrol agent (Takeuchi *et al.*, 2014). Therefore, we hypothesized that *P. putida* did not essentially have effective antifungal activity.

### Specific *P. putida* group strains harbor a quorum-sensing system

Some *P. putida* strains produce AHL as a quorum-sensing signaling compound, and various phenotypes are controlled by AHL-mediated quorum sensing (Steidle *et al.*, 2002; Bertani and Venturi, 2004; Dubern *et al.*, 2006). Therefore, AHL production by *P. putida* strains was assayed using the cross-streaking method with the AHL reporter strains CV026 and VIR07, which respond to short acyl-chain AHL (C ≤8) and long acyl-chain AHL (C ≥8), respectively (Table 1). The results of the AHL production assay revealed that three OTU7 strains, two OTU4 strains, and three singletons produced AHL. All three strains belonging to OTU7 produced AHL; therefore, the whole-genome shotgun sequencing of one of the OTU7 strains, JCM 20066, was performed using the HiSeq X Ten platform (Table 2). JCM 20066 comprises a quorum-sensing system consisting of the AHL synthesis gene *ppuI* (PPUJ20066_41380), transcriptional regulatory gene *rsaL* (PPUJ20066_41370), and AHL receptor gene *ppuR* (PPUJ20066_41360). The *ppuI*-*rsaL*-*ppuR* quorum-sensing system has been also detected in *P. putida* PCL1445 (Dubern *et al.*, 2006), *P. putida* IsoF (Steidle *et al.*, 2002), *P. putida* WCS358 (Bertani and Venturi, 2004), and *Pseudomonas* sp. StFLB209 (Kato *et al.*, 2015). The LC-MS/MS ana­lysis revealed that JCM 20066 produced 3-oxo-C10-HSL as a quorum-sensing signaling compound as well as the three *Pseudomonas* strains described above (Fig. S3). To elucidate the phenotype controlled by 3-oxo-C10-HSL-mediated quorum sensing, we constructed a deletion mutant of the *ppuI* and *ppuR* genes in JCM 20066. AHL production and motility completely disappeared, and biofilm formation clearly decreased in both mutants. The addition of 1‍ ‍μM 3-oxo-C10-HSL restored the motility of and biofilm formation by the *ppuI* mutant, but not by the *ppuR* mutant (Fig. S4). Therefore, 3-oxo-C10-HSL produced by PpuI may bind to the PpuR receptor protein and the PpuR-3-oxo-C10-HSL complex may activate the transcription of genes involved in motility and biofilm formation.

### Comparative genome ana­lysis of *P. putida* strains

Since marked differences were not observed in the phenotypes of OTU4 strains that may be identified as true *P. putida*, a comparative genome ana­lysis was performed to cluster the seven OTU4 strains using whole-genome similarity. The whole-genome shotgun sequencing of the seven OTU4 strains and related strains was performed using the NovaSeq 6000 platform (Table 2). Whole-genome similarity with *P. putida* type strain NBRC 14164^T^ (=JCM 13063^T^) was calculated using ANI and dDDH values. ANI ≥95% and dDDH ≥70% are classified as the same species (Goris *et al.*, 2007; Srivastava *et al.*, 2020). Based on the results of calculations of the ANI and dDDH values of *P. putida* strains against the genome sequence of the *P. putida* type strain, three strains, JCM 20005, JCM 21368, and JCM 13061, were identified with the same species as that of the *P. putida* type strain (Table 2). The other three OTU4 strains, JCM20187, JCM 20188, and JCM 14351, did not meet this criterion (ANI <95% and dDDH ≤60%). Therefore, these strains may be characterized as different from true *P. putida*. Furthermore, since five strains belonged to OTU3 or OTU5, and singletons (NBRC 110474) showed low ANI (≤90%) and dDDH (≤45%) values, these strains belonged to phylogenetically different species from *P. putida*. Since the OTU4 strains were divided into two slightly different branches in the phylogenetic tree (Fig. 1), true *P. putida* may be classified using minor differences in the concatenated sequences of the 16S rRNA and *rpoD* genes.

### Development of specific PCR primers for the identification of true *P. putida*

**Fig. 2.**
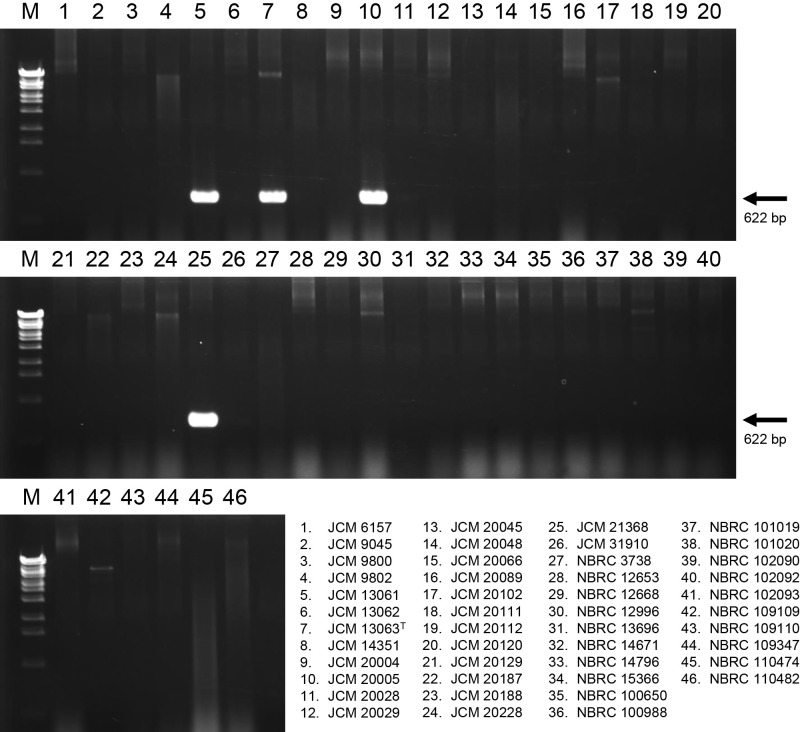
Specificity testing of primers designed from the sequence of PP4_28660 by agarose gel electrophoresis. PCR products were electrophoresed on 1.5% agarose gel. Lane M, One Step Marker 6 (Nippon Gene) used for the DNA size marker. Lane 1–46, *Pseudomonas putida* strains used for the PCR template described at the bottom of the picture.

To identify common genes among true *P. putida* strains, orthologous genes present in only four true *P. putida* strains were searched using the amino acid sequences of the CDSs identified from the whole-genome shotgun sequences listed in Table 2. Twenty-six orthologous genes were identified. The whole-genome sequences of 257 strains of *P. putida* have been deposited on the NCBI Genome website as of January 20, 2023. Twenty-nine genome sequences that showed high ANI values with the genome sequence of the *P. putida* type strain were selected (Table S3). The presence of 26 orthologous genes in the 29 genome sequences was assessed using the BLAST program (Altschul *et al.*, 1990). Seven orthologous genes were highly conserved among the genome sequences that showed high ANI and dDDH values with those of *P. putida* JCM 13063^T^ (Table S3). One of these, PP4_28660, derived from the complete genome sequence of *P. putida* JCM 13063^T^, was selected as the target ortholog. PP4_28660 comprised 639 bp, encoded a hypothetical protein, and was present in all genome sequences that showed high genome similarity (ANI ≥95% and dDDH ≥70) with that of the *P. putida* type strain. Based on the DNA sequences of PP4_28660 and its homolog in the *P. putida* genome, specific primers for the amplification of the internal region of the PP4_28660 homolog were designed (Table S1). To evaluate the potential use of these specific primers for the selection of true *P. putida*, we amplified the PP4_28660 partial region by direct colony PCR. The results obtained showed that a 622-bp PCR fragment of PP4_28660 was successfully amplified from the colony of only four strains, JCM 13063^T^, JCM 13061, JCM 20005, and JCM 21368 showing high genome similarity (ANI ≥95% and dDDH ≥70) with that of the *P. putida* type strain (Fig. 2). These results demonstrate that specific primers for the amplification of the PP4_28660 homolog are useful for selecting only true *P. putida* strains from miscellaneous bacterial isolates.

### Conclusion

We herein demonstrated that 46 strains of *P. putida* obtained from Japanese culture collections may be classified into multiple OTUs based on the concatenated sequences of the 16S rRNA and *rpoD* genes. Only four strains may be identified as true *P. putida*, while the other 42 strains may be reclassified as other species or subspecies of the genus *Pseudomonas*. Historically, numerous *P. putida* strains have been isolated, characterized, and utilized in various fields. For example, *P. putida* KT2440 is a well-known *P. putida* strain that was isolated in the 20th century (Regenhardt *et al.*, 2002), and is expected to be used as a biocontrol and bioremediation agent (Matilla *et al.*, 2010; Gong *et al.*, 2017). However, the complete genome sequence of strain KT2440 (accession no. AE015451) showed lower ANI (89.85%) and dDDH (40.60%) values against those of *P. putida* JCM 13063^T^. Therefore, most of the bacterial strains classified as *P. putida* globally may be essentially different species from *P. putida*. While difficulties are associated with establishing whether a large number of *P. putida* strains may be classified as true *P. putida* using traditional classification methods, the newly developed primers in the present study render the identification of *P. putida* easier. However, *P. putida* isolates, which are potentially different from true *P. putida*, included specific bacterial groups with unique phenotypes, such as OTU7, which contains the *ppuI-rsaL-ppuR* quorum-sensing system. In the future, a comparative genome ana­lysis of *P. putida* strains that have already been roughly classified may enable the identification of novel *Pseudomonas* species with unique phenotypes.

## Citation

Morohoshi, T., Yaguchi, N., and Someya, N. (2023) Genomic Reclassification and Phenotypic Characterization of *Pseudomonas putida* Strains Deposited in Japanese Culture Collections. *Microbes Environ ***38**: ME23019.

https://doi.org/10.1264/jsme2.ME23019

## Supplementary Material

Supplementary Material

## Figures and Tables

**Table 1.** OTU classification and phenotypic features of *Pseudomonas putida* strains used in the present study.

Strains	OTU^a^	AHL production^b^		Motility^c^	Fluorescence	Antifungal activity
		CV026	VIR07			
JCM 9802	1	–	–	++	+	–
JCM 9800	1	–	–	+	+	–
JCM 9045	1	–	–	–	+	–
JCM 31910	1	–	–	–	+	–
JCM 20048	1	–	–	+	+	–
JCM 20045	1	–	–	–	+	–
JCM 20004	1	–	–	+	+	–
NBRC 12668	1	–	–	++	+	–
NBRC 109110	1	–	–	–	+	–
NBRC 109109	1	–	–	–	+	–
NBRC 101019	1	–	–	+	+	–
NBRC 100988	1	–	–	++	+	–
NBRC 100650	1	–	–	–	+	–
JCM 6157	2	–	–	–	+	–
JCM 20129	2	–	–	+	+	–
JCM 20089	2	–	–	++	+	–
JCM 20028	3	–	–	+	+	–
NBRC 14671	3	–	–	++	+	–
JCM 20188	4	–	–	–	+	–
JCM 20187	4	–	+	–	–	–
JCM 20005	4	–	–	+	+	–
JCM 14351	4	–	+	–	+	–
JCM 13063^T^	4	–	–	–	+	–
JCM 13061	4	–	–	–	+	–
JCM 21368	4	–	–	–	+	–
NBRC 15366	5	–	–	++	+	–
NBRC 12996	5	–	–	+	+	–
NBRC 109347	5	–	–	++	+	+
JCM 20228	6	–	–	++	+	–
JCM 20029	6	–	–	–	+	–
JCM 20112	7	–	++	–	–	–
JCM 20102	7	–	++	++	–	–
JCM 20066	7	–	++	++	+	–
JCM 20111	8	–	–	+	+	–
NBRC 101020	9	–	–	++	+	–
NBRC 3738	ST	–	++	–	–	–
NBRC 14796	ST	++	+	++	+	+
NBRC 13696	ST	–	–	–	+	–
NBRC 12653	ST	–	–	+	+	–
NBRC 110482	ST	–	+	++	+	–
NBRC 110474	ST	–	–	+	+	–
NBRC 102093	ST	–	–	++	+	–
NBRC 102092	ST	–	–	++	+	–
NBRC 102090	ST	–	–	–	+	–
JCM 20120	ST	–	–	–	+	–
JCM 13062	ST	–	–	–	+	–

^a^ST, singleton; ^b^AHL production means strong (++), weak (+), or no (–) induction of violacein production in AHL reporter strains; ^c^Diameter of the spread colony after a 24-h incubation, ≥15‍ ‍mm (++) >5‍ ‍mm (+), or no motility (–).

**Table 2.** Genomic relationships between *Pseudomonas putida* strains and *P. putida* type strain NBRC 14164^T^.

Strains	OTU^a^	ANI (%)^b^	dDDH (%)^b^	Genome information			
				Total size (bp)	GC cont. (%)	CDSs	Accession no.^c^
JCM 13063^T^ (=NBRC 14164^T^)	4	100	100	6,156,701	62.3	5,372	AP013070*
JCM 20005	4	99.4	95.6	6,055,601	62.3	5,387	BSKE01000000
JCM 21368	4	99.2	94.8	6,321,123	62.3	5,770	BSKH01000000
JCM 13061	4	98.4	87.0	6,473,766	61.8	5,930	BSKD01000000
JCM 20187 (=NRRL B-252)	4	94.9	60.3	6,087,214	62.5	5,364	RWKF00000000*
JCM 14351 (=NRRL B-251)	4	94.8	60.4	6,068,462	62.6	5,352	RWKE00000000*
JCM 20188	4	93.6	54.0	6,201,866	62.1	5,567	BSKG01000000
NBRC 14671	3	90.7	43.6	5,793,231	61.9	5,189	BSKJ01000000
JCM 20028	3	90.7	43.6	5,784,640	61.9	5,188	BSKF01000000
NBRC 110474	ST	90.3	41.5	6,066,026	63.0	5,364	BSKM01000000
NBRC 109347	5	90.1	41.2	5,723,681	63.1	5,162	BSKL01000000
NBRC 12996	5	89.7	40.0	5,796,075	62.6	5,223	BSKI01000000
NBRC 15366	5	89.5	39.5	5,669,176	63.0	5,082	BSKK01000000
JCM 20066	7	86.9	32.8	6,317,210	62.2	5,528	BSKN01000000

^a^ST, singleton; ^b^ANI and dDDH values were calculated using *P. putida* NBRC 14164^T^ as the reference genome; ^c^Asterisks indicate sequences previously deposited in the NCBI database.
