# Supplementary material for: Genomic Reclassification and Phenotypic Characterization of Pseudomonas putida Strains Deposited in Japanese Culture Collections
Source: Microbes Environ. 2023 Jun 6;38(2):ME23019. doi: 10.1264/jsme2.ME23019 (PMC10308231; doi:10.1264/jsme2.ME23019)
Supplement: Supplementary file 1 — Supplementary Material [file 38_23019_s1.pdf]

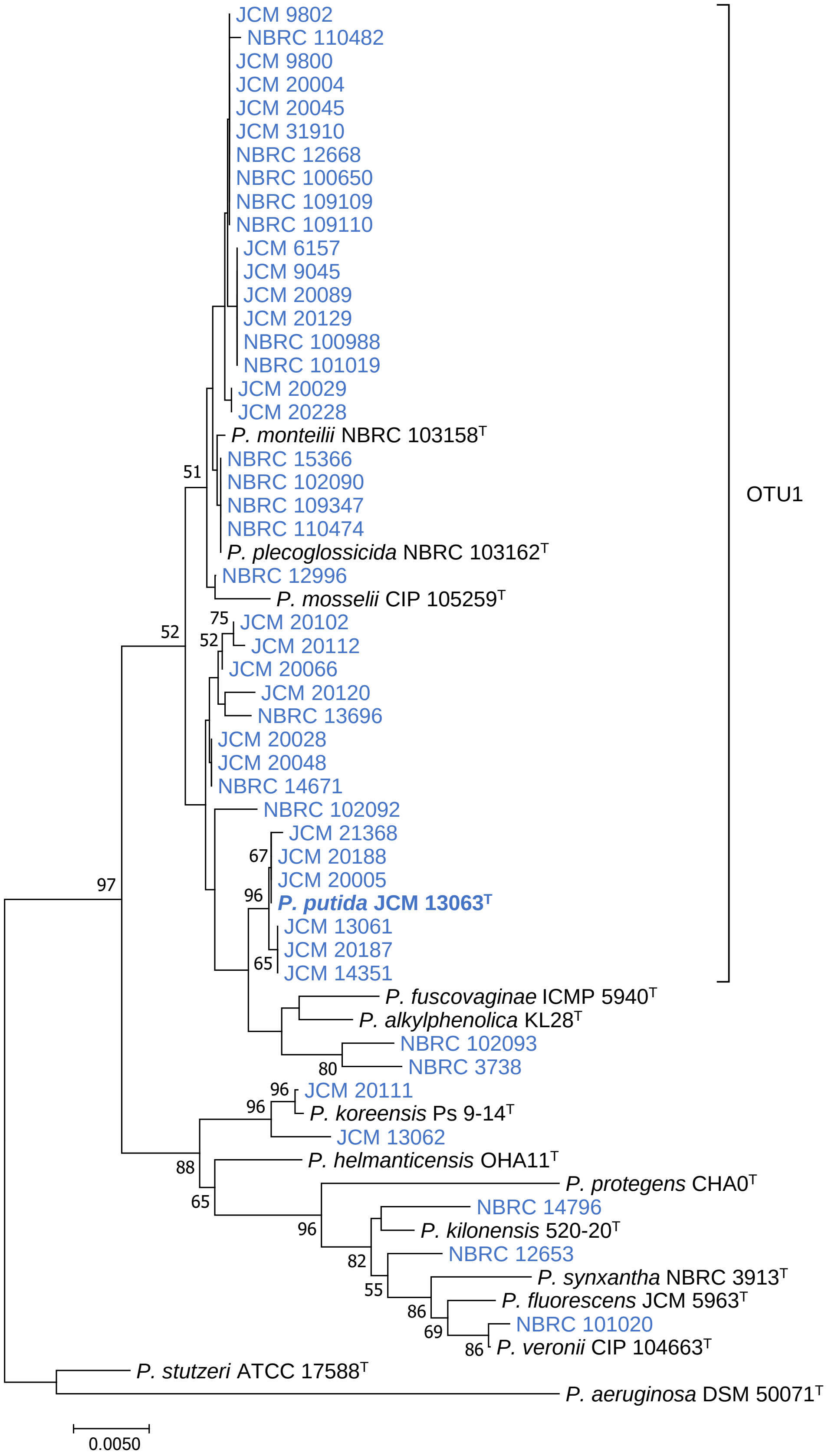

Fig. S1. Phylogenetic tree based on 16S rRNA sequences of *P. putida* strains (blue) and type strains of other *Pseudomonas* species (black). The *P. putida* type strain JCM 13063^{T} is shown in bold. A phylogenetic tree was constructed using the neighbor-joining method with ClustalW in the MEGA 7 program. The percentage of replicate trees in which the associated taxa clustered together in the bootstrap test (500 replicates) is shown next to the branches. The scale bar represents 0.005 substitutions per nucleotide position.

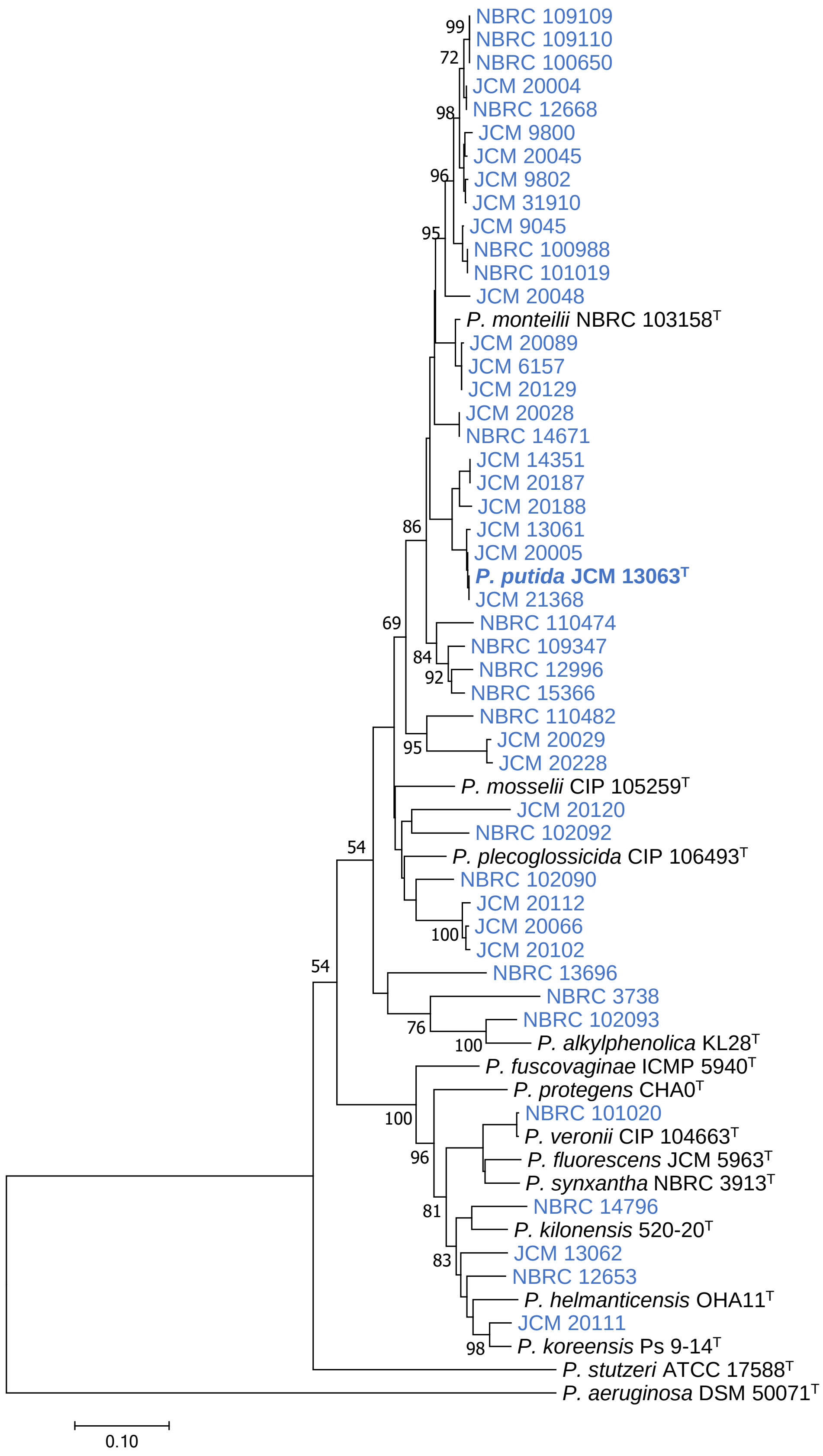

Fig. S2. Phylogenetic tree based on the *rpoD* gene sequences of *P. putida* strains (blue) and type strains of other *Pseudomonas* species (black). The *P. putida* type strain JCM 13063^{T} is shown in bold. A phylogenetic tree was constructed using the neighbor-joining method with ClustalW in the MEGA 7 program. The percentage of replicate trees in which the associated taxa clustered together in the bootstrap test (500 replicates) is shown next to the branches. The scale bar represents 0.1 substitutions per nucleotide position.

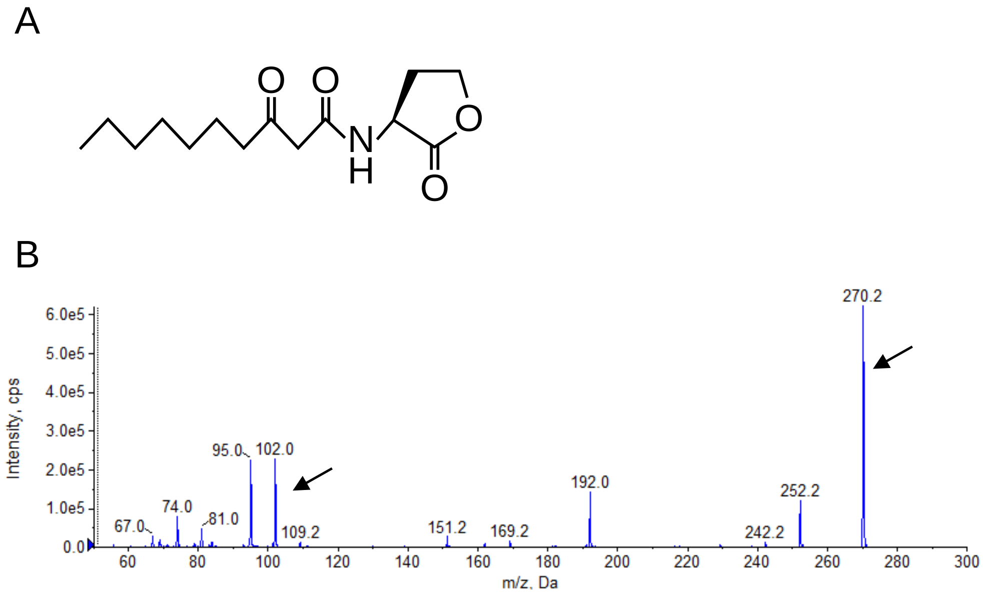

Fig. S3. (A) The structure of 3-oxo-C10-HSL. (B) Mass spectrum of 3-oxo-C10-HSL extracted from the cell-free supernatant of *P. putida* JCM 20066. After fractionation using reverse-phase HPLC, the ESI-MS/MS fragment peaks of the AHLs were analyzed. The corresponding peaks for 3-oxo-C10-HSL (*m/z* 270) and the product ion peaks (*m/z* 102) are marked by arrows.

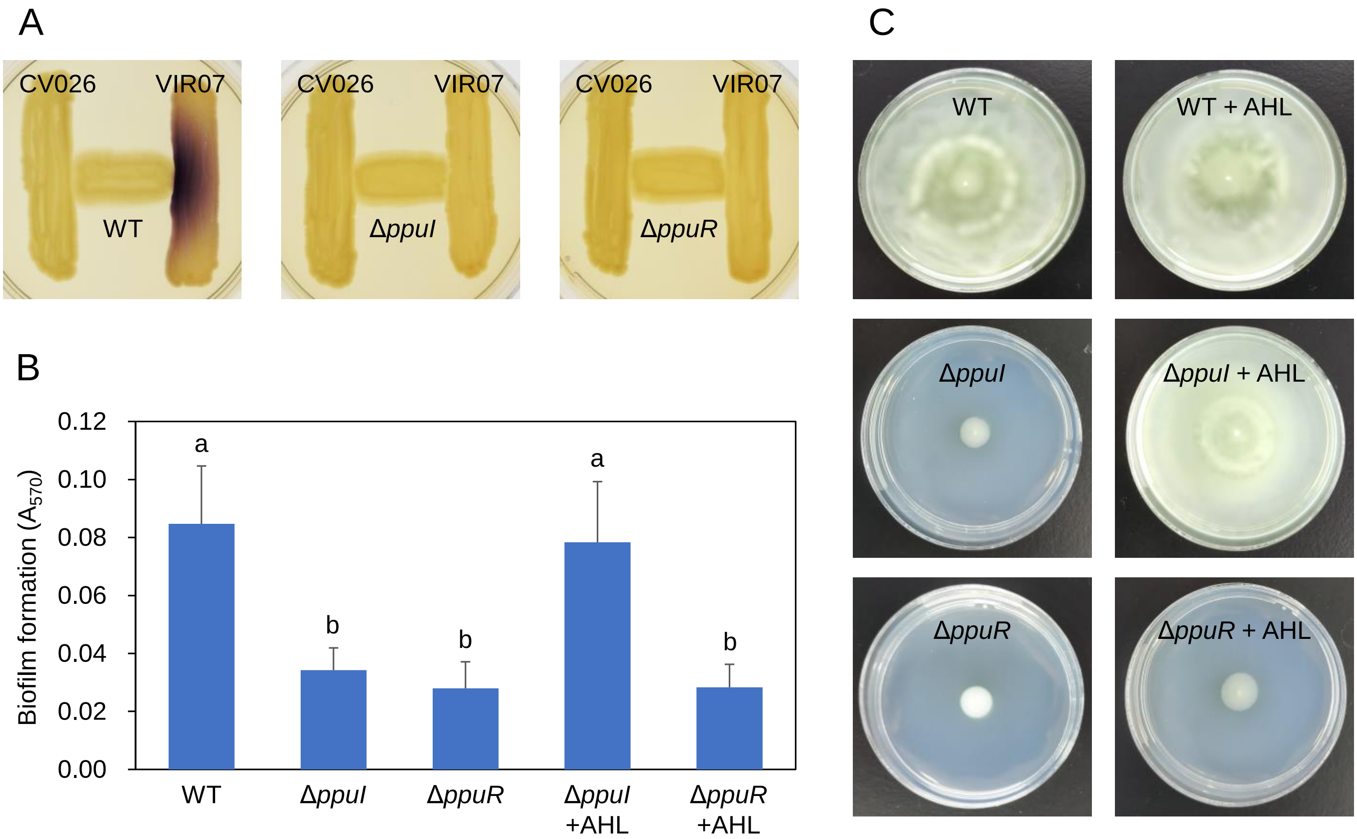

Fig. S4. Characterization of *P. putida* JCM 20066 wild type strain (WT) and its mutants. AHL in the figure refers to the addition of 3-oxo-C10-HSL at the final concentration of 1 µM. (A) Cross-streak assay for AHL detection. CV026 and VIR07 were vertically streaked on each TSB agar plate, whereas test strains were horizontally streaked between CV026 and VIR07. After overnight incubation at 30 °C, AHL was detected as production of the purple pigment violacein. (B) Quantification of biofilm formed by JCM 20066 and its mutants. Ten samples were averaged, and error bars indicate standard deviations. Different letters above the bars indicate significant differences among treatments according to Tukey's HSD test ($P<0.01$). (C) Motility of JCM 20066 and its mutants. Strains were inoculated at the center of KB medium containing 0.5 wt% agar and incubated at 30°C for 24 h.

Table S1. Primers used in this study

Description	Primers	Primer sequences (5'-3')*
Amplification of 16S rRNA gene	16SF3	CTCAACTTGAGAGTTTGATCCTGGCTCAG
	16SR3	CCAGCCGCAGGTTCYCCTACGGCTACCTTG
Amplification of *rpoD*	PsEG30F	ATYGAAATCGCCAARCG
	PsEG790R	CGGTTGATKTCCTTGA
Amplification of PP4_28660	Ppu28660-F	CATTTAGAGCGTTTGCGGTTGTCGGTCTCTGTTCG
	Ppu28660-R	CCATAGTAGAAACCCTCTGCAGTAAGCGCCACAG
Amplification of *ppuI* from JCM 20066	ppuI-F	GCAAGACCACCTGGGAAATCTCGATGATCTTGTCG
	ppuI-R	CAGCGCCAGTGAACTGATTTTCCGCTCCATCG
Amplification of *ppuR* from JCM 20066	ppuR-F	CAACCATGCTGAAGAACTGTTGCTGGAACATGCAG
	ppuR-R	CGTCATCGCGGTTGATGATCATGTAATAGGGGTCG
Deletion of *ppuI* internal sequence	ppuIdel-F	TCT<u>GGATCC</u>CGTCGAGAAGATGTTGATTCGCCTGG
	ppuIdel-R	TCT<u>GGATCC</u>CCATGTCATTGATCAGTGGGATGTCC
Deletion of *ppuR* internal sequence	ppuRdel-F	TCT<u>GGATCC</u>CTTCAAGAACATCCGCCGCAAGTTCC
	ppuRdel-R	TCT<u>GGATCC</u>CAATGCTGAACAACCGGGTCGACTGC

**Bam*HI sites are underlined.

Table S2. Accession numbers of 16S rRNA and *rpoD* sequences derived from *P. putida* and type strains of other *Pseudomonas* species.

Species	Strains	Accession no.	
		16S rRNA	*rpoD*
P. putida	JCM 6157	AF094746	LC752188*
	JCM 9045	LC752229*	LC752189*
	JCM 9800	LC752230*	LC752190*
	JCM 9802	D87102	BBDA00000000**
	JCM 13061	AF094745	AB039580
	JCM 13062	AF095892	LC752191*
	JCM 13063^T	AF094736	AB039581
	JCM 14351	RWKE00000000**	RWKE00000000**
	JCM 20004	LC571911	LC752192*
	JCM 20005	LC571912	LC752193*
	JCM 20028	LC571920	LC752194*
	JCM 20029	LC571921	LC752195*
	JCM 20045	LC571924	LC752196*
	JCM 20048	LC571927	LC752197*
	JCM 20066	LC571932	LC752198*
	JCM 20089	LC571940	LC752199*
	JCM 20102	LC595301	LC752200*
	JCM 20111	LC595303	LC752201*
	JCM 20112	LC595304	LC752202*
	JCM 20120	LC595308	LC752203*
	JCM 20129	LC595310	LC752204*
	JCM 20187	RWKF00000000**	RWKF00000000**
	JCM 20188	LC595320	LC752205*
	JCM 20228	LC654885	LC752206*
	JCM 21368	LC752231*	LC752207*
	JCM 31910	L37365	LC752208*
	NBRC 3738	AB680123	LC752209*
	NBRC 12653	AB680304	LC752210*
	NBRC 12668	AB680312	LC752211*
	NBRC 12996	AB680362	LC752212*
	NBRC 13696	AB680483	LC752213*
	NBRC 14671	AB680647	LC752214*
	NBRC 14796	AB680667	LC752215*
	NBRC 15366	AB680847	LC752216*
	NBRC 100650	AB681214	LC752217*
	NBRC 100988	AB681323	LC752218*
	NBRC 101019	AB681332	LC752219*
	NBRC 101020	AB681333	LC752220*
	NBRC 102090	AB681701	LC752221*
	NBRC 102092	AB681703	LC752222*
	NBRC 102093	AB681704	LC752223*
	NBRC 109109	NBRC***	LC752224*
	NBRC 109110	NBRC***	LC752225*
	NBRC 109347	NBRC***	LC752226*
	NBRC 110474	NBRC***	LC752227*
	NBRC 110482	NBRC***	LC752228*
P. fluorescens	JCM 5963^T	LC462170	D86033
P. monteilii	NBRC 103158^T	NR_114224	FN554488
P. plecoglossicida	NBRC 103162^T	NR_114226	FN554503
P. mosselii	CIP 105259^T	AF072688	FN554491
P. alkylphenolica	KL28^T	AY324319	HE577794
P. fuscovaginae	ICMP 5940^T	NR_116700	FN554467
P. koreensis	Ps 9-14^T	NR_025228	FN554476
P. helmanticensis	OHA11^T	NR_126220	HG940517
P. protegens	CHA0^T	NR_114749	CP003190**
P. kilonensis	520-20^T	NR_028929	NR_028929
P. veronii	CIP 104663^T	NR_028706	FN554518
P. synxantha	NBRC 3913^T	AB680171	JN589943
P. stutzeri	ATCC 17588^T	AF094748	KR780035
P. aeruginosa	DSM 50071^T	X06684	LN831024**

*Deposited in this study; **Genome assembly data; ***Deposited in NBRC Online Catalogue

Table S3. Distribution of orthologous genes in whole-genome sequences of *P. putida* strains

Strains	ANI[a] (%)	dDDH[a] (%)	Orthologues[b]								Accession no.
			PP4_28660 639 bp	PP4_36480 183 bp	PP4_02740 440 bp	PP4_02730 273 bp	PP4_46100 549 bp	PP4_36770 807 bp	PP4_36760 957 bp	PP4_00010[c] 1545 bp	
JCM 13063^{T} (=NBRC 14164^{T})	100	100	+	+	+	+	+	+	+	+	AP013070
IPO3752	99.96	99.90	+	+	+	+	+	+	+	+	JACAQQ000000000
JCM 20005	99.43	95.60	+	+	+	+	+	+	+	+	BSKE01000000
JCM 21368	99.24	94.80	+	+	+	+	+	+	+	+	BSKH01000000
UMG622	98.91	90.10	+	+	+	+	+	+	+	+	SUPX00000000
UME3119	98.85	90.10	+	+	+	+	+	+	+	+	SUQE00000000
UME3142	98.85	90.00	+	+	+	+	+	+	+	+	SUQD00000000
UME3145	98.84	90.00	+	+	+	+	+	+	+	+	SUQC00000000
UME3100	98.82	90.10	+	+	+	+	+	+	+	+	SUQF00000000
UMG610	98.82	90.00	+	+	+	+	+	+	+	+	SUPZ00000000
UMG604	98.80	90.10	+	+	+	+	+	+	+	+	SUQB00000000
UMG605	98.79	90.10	+	+	+	+	+	+	+	+	SUQA00000000
UMG612	98.79	90.20	+	+	+	+	+	+	+	+	SUPY01000000
Z13	98.75	90.90	+	+	+	+	+	+	+	+	JAINFM000000000
PSB00023	98.42	87.00	+	+	+	+	+	+	+	+	JADUCH000000000
HAMBI_6	98.37	86.90	+	+	+	+	+	+	+	+	QLLF00000000
JCM 13061	98.36	87.00	+	+	+	+	+	+	+	+	BSKD01000000
DZ-C20	98.36	86.90	+	+	+	+	+	+	+	+	NBWB00000000
PSB00025	98.35	86.70	+	+	+	+	+	+	+	+	JADUCG000000000
791_PPUT	98.35	86.60	+	+	+	+	+	+	+	+	NZ_JUST00000000
JCM 18452	98.31	86.00	+	+	+	+	+	+	+	+	BBDB00000000
PSB00015	97.86	82.20	+	+	+	+	+	–	–	+	JADUCN000000000
JQ581	97.26	76.60	+	+	–	–	–	+	+	+	CP050951
JCM 20187 (=NRRL B-252)	94.91	60.30	–	–	–	–	–	–	–	+	RWKF00000000
JCM 14351 (=NRRL B-251)	94.84	60.40	–	–	–	–	–	–	–	+	RWKE00000000
JUb85	94.76	60.20	–	–	–	–	–	–	–	+	SLXX00000000
S13.1.2	94.69	60.40	–	–	–	–	–	–	–	+	CP010979
W15Oct28	94.44	59.10	–	–	–	–	+	–	–	+	JENB00000000
JCM 20188	93.56	54.00	–	–	–	–	–	–	–	+	BSKG01000000

[a]ANI and dDDH values were calculated using *P. putida* NBRC 14164^{T} as the reference genome; [b]Orthologous genes are described as locus tags of the complete genome of NBRC 14164^{T}; [c]PP4_00010 (*dnaA*) was selected as a common gene.
